# Distinctive phosphorylation pattern during mitotic exit network (MEN) regulation is important for the development and pathogenicity of *Magnaporthe oryzae*

**DOI:** 10.1007/s44154-022-00063-0

**Published:** 2022-09-20

**Authors:** Wanzhen Feng, Jiansheng Wang, Xinyu Liu, Haowen Wu, Muxing Liu, Haifeng Zhang, Xiaobo Zheng, Ping Wang, Zhengguang Zhang

**Affiliations:** 1 Department of Plant Pathology, College of Plant Protection, Nanjing Agricultural University, and Key Laboratory of Integrated Management of Crop Diseases and Pests, Ministry of Education, Nanjing, 210095 China; 2 The Key Laboratory of Plant Immunity, Nanjing Agricultural University, Nanjing, 210095 China; 3 Plant Protection and Quarantine Station of Nanjing, Nanjing, 210095 China; 4 Departments of Microbiology, Immunology, and Parasitology, and Pediatrics, Louisiana State University Health Sciences Center, New Orleans, Louisiana 70112 USA

**Keywords:** Mitotic exit network (MEN), Phosphorylation, Pathogenicity, Rice blast

## Abstract

The mitotic exit network (MEN) pathway is a vital kinase cascade regulating the timely and correct progress of cell division. In the rice blast fungus *Magnaporthe oryzae*, the MEN pathway, consisting of conserved protein kinases MoSep1 and MoMob1-MoDbf2, is important in the development and pathogenicity of the fungus. We found that deletion of *MoSEP1* affects the phosphorylation of MoMob1, but not MoDbf2, in contrast to what was found in the buddy yeast *Saccharomyces cerevisiae*, and verified this finding by in vitro phosphorylation assay and mass spectrometry (MS) analysis. We also found that S43 residue is the critical phosphor-site of MoMob1 by MoSep1, and proved that MoSep1-dependent MoMob1 phosphorylation is essential for cell division during the development of *M. oryzae*. We further provided evidence demonstrating that MoSep1 phosphorylates MoMob1 to maintain the cell cycle during vegetative growth and infection. Taken together, our results revealed that the MEN pathway has both distinct and conservative functions in regulating the cell cycle during the development and pathogenesis of *M. oryzae*.

## Introduction

The cell cycle is a complex and ubiquitous process involving numerous regulatory proteins during the growth and development of eukaryotes(Schafer 1998). The cores of this process are cyclin-dependent kinases (Cdks) and the cyclin proteins, which guide cells through specific event sequences, eventually leading to mitosis and the production of two daughter cells (MacKenzie and Lacefield 2020). As a periodic and continuous process (Coffman 2004; Hoyt et al. 1991)，it is necessary to complete the replication of chromosomal genetic materials during mitosis (Bolcun-Filas and Handel 2018; Bolcun-Filas and Schimenti 2012), including nuclear membrane cleavage, release of genomic genetic materials into the cytoplasm (Bzymek et al. 2010; Colicino and Hehnly 2018; Daniel et al. 1998), and the distribution of chromosomal genetic materials and the cytoplasm to the daughter cells to form somatic cells with the same chromosomal genetic materials (Arbel-Eden and Simchen 2019; Kapoor 2017). In the process of cell division, each stage will be precisely regulated by specific proteins to ensure the orderly progress of mitosis (Argunhan et al. 2018; Bizard and Hickson 2018; Bolcun-Filas and Handel 2018). The mitotic entry governed by Cyclin B (Cyc B)-Cdk1 (Hégarat et al. 2016; Nurse 1990), which co-operates with other mitotic kinases to induce a burst of protein phosphorylation to control mitotic events (Ma and Poon 2011). And then the activated Cdc14 is released from the nucleus into the cytoplasm leading to the mitotic exit process (Hershko 1999; Musacchio 2015; Stukenberg and Burke 2015).

In *Saccharomyces cerevisiae*, the spindle position checkpoint (SPOC) curbs mitotic exit and cytokinesis until the mitotic spindle is properly oriented to ensure the normal generation of progeny cells (Fraschini et al. 2008). The small GTPase Tem1 functioning as the central switch to regulate mitotic exit network (MEN) activity is the target of the SPOC (Caydasi et al. 2010; Scarfone and Piatti 2015). The GTPase-activating complex Bub2-Bfa1 keeps Tem1 in the GDP-bound inactive form (Fraschini et al. 2008; Shirayama et al. 1994; Visintin and Amon 2001). During the late anaphase, Tem1-GTP is thought to bind to the spindle pole body (SPB) and activate the protein kinase Cdc15, which in turn activates the downstream kinase Dbf2 associated with its activating subunit, Mob1 (Maekawa et al. 2007; Mohl et al. 2009; Rock and Amon 2011). The Dbf2-Mob1 kinase complex Dbf2-Mob1 further phosphorylates Cdc14, leading to its release from the nucleolus to regulate the completion of cell division (Hershko 1999; Musacchio 2015; Stukenberg and Burke 2015).

*Magnaporthe oryzae* causes devastating blast on rice (Ebbole 2007; Zhang et al. 2016). The conidia of *M. oryzae* germinate and form specialized infection structures called appressoria that breach the leaf cuticle and expand inside the host (Fernandez and Orth 2018; Liu and Lin 2008). Conidia germination requires not only the recognition of physical signals, such as hardness and hydrophobicity but also the correct cell cycle system (Dagdas et al. 2012; Zhang et al. 2016). During germination, cells that produce the germ tube will undergo mitosis, and the newly produced nuclei would enter the appressoria from the germ tubes. During appressorial maturation, three nuclei in conidia would gradually degrade, leaving only one nucleus in the appressorium that transferred to the invading nail with the germ tube. It then undergoes multiple rounds of division during the expansion of invading hypha (Fukada et al. 2019; Li et al. 2018; Saunders et al. 2010). Therefore, the tightened regulation of cell division is essential for appressorium formation in *M. oryzae*.

In *M. oryzae*, previous studies showed that the deletion of *MoBUB2* and *MoBFA1* homologs affects the proper switch from apical to polar growth because of the shorter G1 phase duration (Fukada et al. 2019). Meanwhile, we previously identified three key proteins, MoSep1, MoDbf2, and MoMob1, of the MEN pathway, necessary for normal life activities*.* Deletion of these genes, individually, resulted in increased septation and nuclear division in the germ tubes and defects in appressorium formation and infection (Feng et al. 2021). Results of the phosphorylation assay showed that phosphorylation levels of MoDbf2 in the Δ*Μosep1* mutant were not significantly different from the wild type. This is different from the phosphorylation modification of Dbf2 by Cdc15/Sep1 in *S. cerevisiae*. Because of the difference, we hypothesized that distinctions in functional mechanisms of the MEN pathway might exist between *S. cerevisiae* and *M. oryzae*.

## Results

### MoSep1 interacts with MoDbf2 and MoMob1

**Fig. 1:**
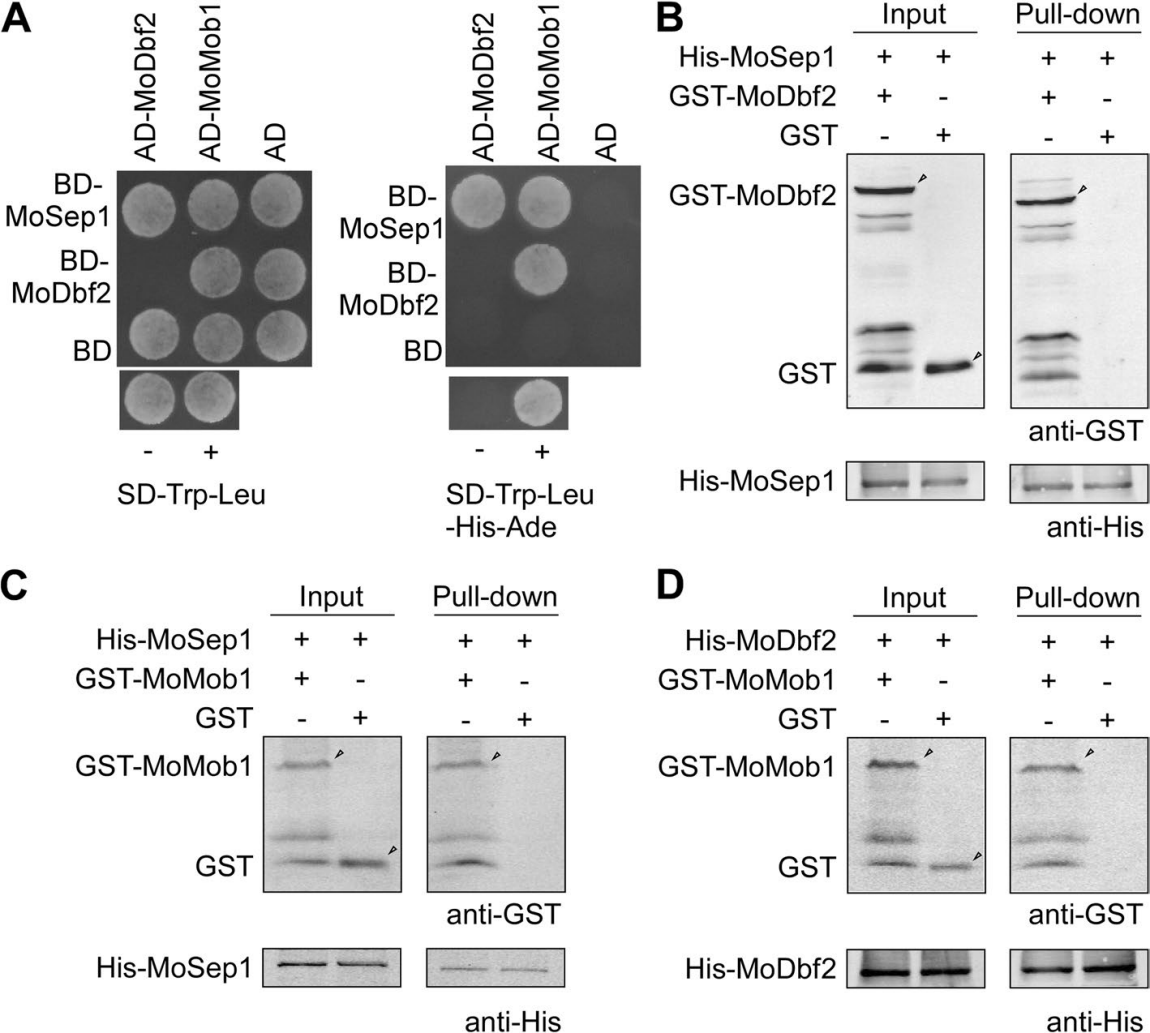
MoSep1 interacts with MoDbf2 and MoMob1. **A** Yeast two-hybrid analysis of the interaction between MoMSep1 and MoDbf2. MoMob1 was inserted into vectors pGADT7 and pGBKT7. BD-MoMSep1 and AD-MoDbf2, BD-MoMSep1 and AD-MoMob1, and BD-MoDbf2 and AD-MoMob1 were co-introduced into the yeast AH109 strain and then incubated on SD-Leu-Trp (as control) and SD-Leu-Trp-His-Ade (for selection) for 5 days. MoSep1, MoDbf2, and MoMob1 interacted with each other. **B** In vitro pull-down assay between His-MoSep1 and GST-MoDbf2. Recombinant His-MoSep1 bound to histidine beads was incubated with bacterial cell lysate containing GST-MoDbf2 or GST, respectively. Eluted protein was analyzed by immunoblot (IB) with monoclonal anti-His and monoclonal anti-GST antibodies. MoSep1 interact with MoDbf2. **C** In vitro pull-down assay between His-MoSep1 and GST-MoMob1. Recombinant His-MoSep1 bound to histidine beads was incubated with bacterial cell lysate containing GST-MoMob1 or GST, respectively. Eluted protein was analyzed by immunoblot (IB) with monoclonal anti-His and monoclonal anti-GST antibodies. MoSep1 interact with MoMob1. **D** In vitro pull-down assay between His-Dbf2 and GST-MoMob1. Recombinant His-MoDbf2 bound to histidine beads was incubated with bacterial cell lysate containing GST-MoMob1 or GST, respectively. Eluted protein was analyzed by immunoblot (IB) with monoclonal anti-His and monoclonal anti-GST antibodies. MoDbf2 interact with MoMob1. His, histidine. GST, glutathione transferase

Previous studies indicated that Δ*Μosep1*, Δ*Μodbf2*, and Δ*Μomob1* mutants are defective in mitotic exit. Nuclei division in these mutants is in a non-stop pattern (Feng et al. 2021). To explore the regulatory function of these proteins in mitotic exit, we verified the interaction of MoSep1, MoDbf2, and MoMob1 by yeast-two-hybrid and GST pull-down assays. Results showed that all these three proteins interact with each other (Fig. 1).

### MoSep1 phosphorylates MoMob1

**Fig. 2:**
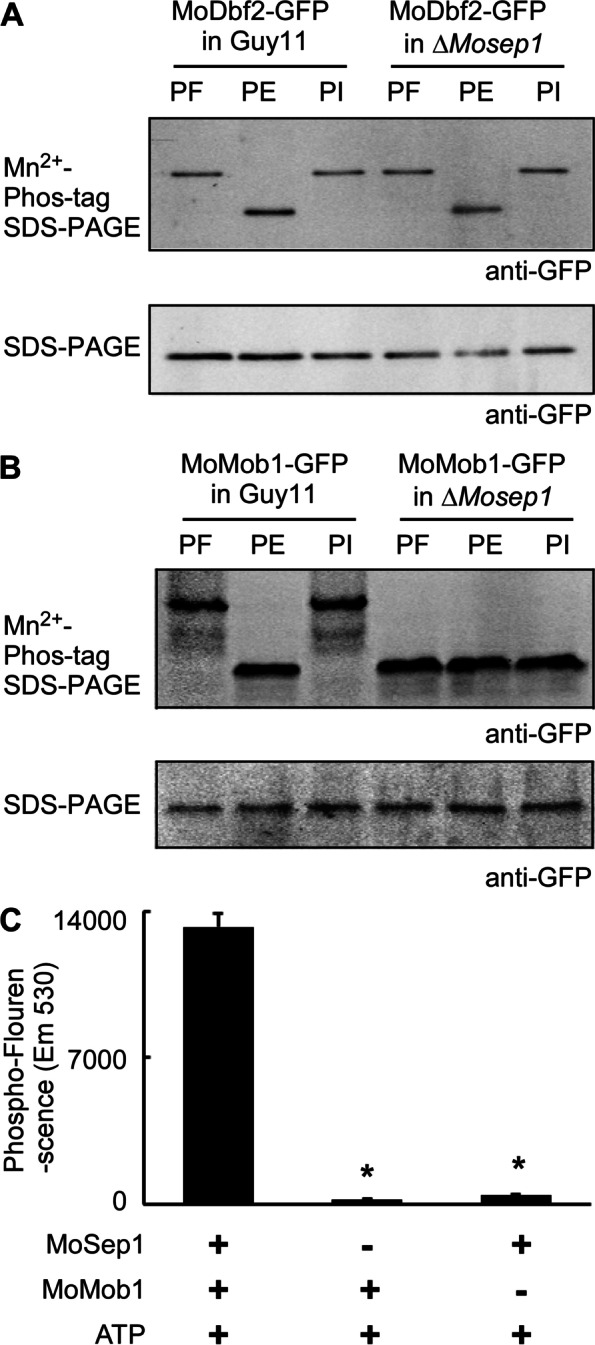
MoSep1 phosphorylates MoMob1. **A** In vivo phosphorylation analysis of MoMob1 in the Guy11 and the Δ*Mosep1* mutant strain. MoMob1-GFP proteins treated with phenylmethylsulfonyl fluorid (PF), phosphatase (PE) and phosphatase inhibitors (PI), and detected by the anti-GFP antibody. The phosphorylated-MoMob1 band is higher than controls when detected in the Mn^2+^-Phos-tag SDS-PAGE gel. **B** In vivo  phosphorylation analysis of MoMob1 in the Guy11 and the Δ*Mosep1* mutant strains. MoMob1-GFP proteins treated with phenylmethylsulfonyl fluorid (PF), phosphatase (PE) and phosphatase inhibitors (PI), and detected by the anti-GFP antibody. The phosphorylated-MoMob1 band is higher than controls when detected in the Mn^2+^-Phos-tag SDS-PAGE gel. **C** In vitro phosphorylation analysis by the fluorescence detection in tube (FDIT) method. Purified proteins of His-MoMob1 and His-MoSep1 were used for kinase reactions in the presence of 50 μm ATP and then dyed with the Pro-Q Diamond Phosphorylation Gel Stain. The fluorescence signal at 590 nm (excited at 530 nm) was measured in a Cytation3 microplate reader (Biotek, Winooski, VT, USA). Error bars represent the standard deviations from three independent experiments. Asterisks indicate statistical significance according to a Student’s test (*p* < 0.01)

In *S. cerevisiae*, Cdc15 (a MoSep1 homolog) phosphorylates Dbf2 to regulate the MEN pathway. To test whether a similar mechanism occurs in *M. oryzae,* we constructed MoMob1-GFP and MoDbf2-GFP fusion genes and introduced them into the wild-type strain Guy11 and the Δ*Μosep1* mutant. We extracted total proteins from Δ*Μosep1/MoDBF2-GFP*, Guy11/*MoDBD2-GFP*, Δ*ΜoSep1/MoMOB1-GFP*, and Guy11/*MoMOB1-GFP*, respectively. We then estimated phosphorylation levels of MoDbf2 and MoMob1 by visualizing migrating patterns of Mn^2+^-Phos-tag SDS-PAGE. The result showed that no significant difference in the band migration rate of MoDbf2-GFP between Guy11 and the Δ*Μosep1* mutant, suggesting that the deletion of *MoSEP1* does not affect MoDbf2 phosphorylation (Fig. 2A). However, the phosphorylated-MoMob1-GFP band (P-MoMob1) was higher than the MoMob1-GFP band (MoMob1) in the presence of a phosphatase inhibitor in Guy11, and the difference disappeared when upon phosphatase treatment. The MoMob1-GFP band in Guy11 was significantly higher than in the Δ*Μosep1* mutant (Fig. 2B). This finding indicated that phosphorylation levels of MoMob1 decreased significantly in the absence of MoSep1. We speculated that MoSep1 might not participate in phosphorylating MoDbf2, or MoDbf2 may also be subjected to phosphorylation by other kinases. Nevertheless, MoSep1 appears as an important kinase for phosphorylating MoMob1.

To confirm MoSep1 directly phosphorylates MoMob1, we constructed His-MoSep1 and His-MoMob1 vectors and performed in vitro phosphorylation assay using protein gel-staining fluorescence dye that showed MoSep1 phosphorylates MoMob1 (Fig. 2C).

### MoMob1 S43 residue is the key phosphorylation site by MoSep1

**Fig. 3:**
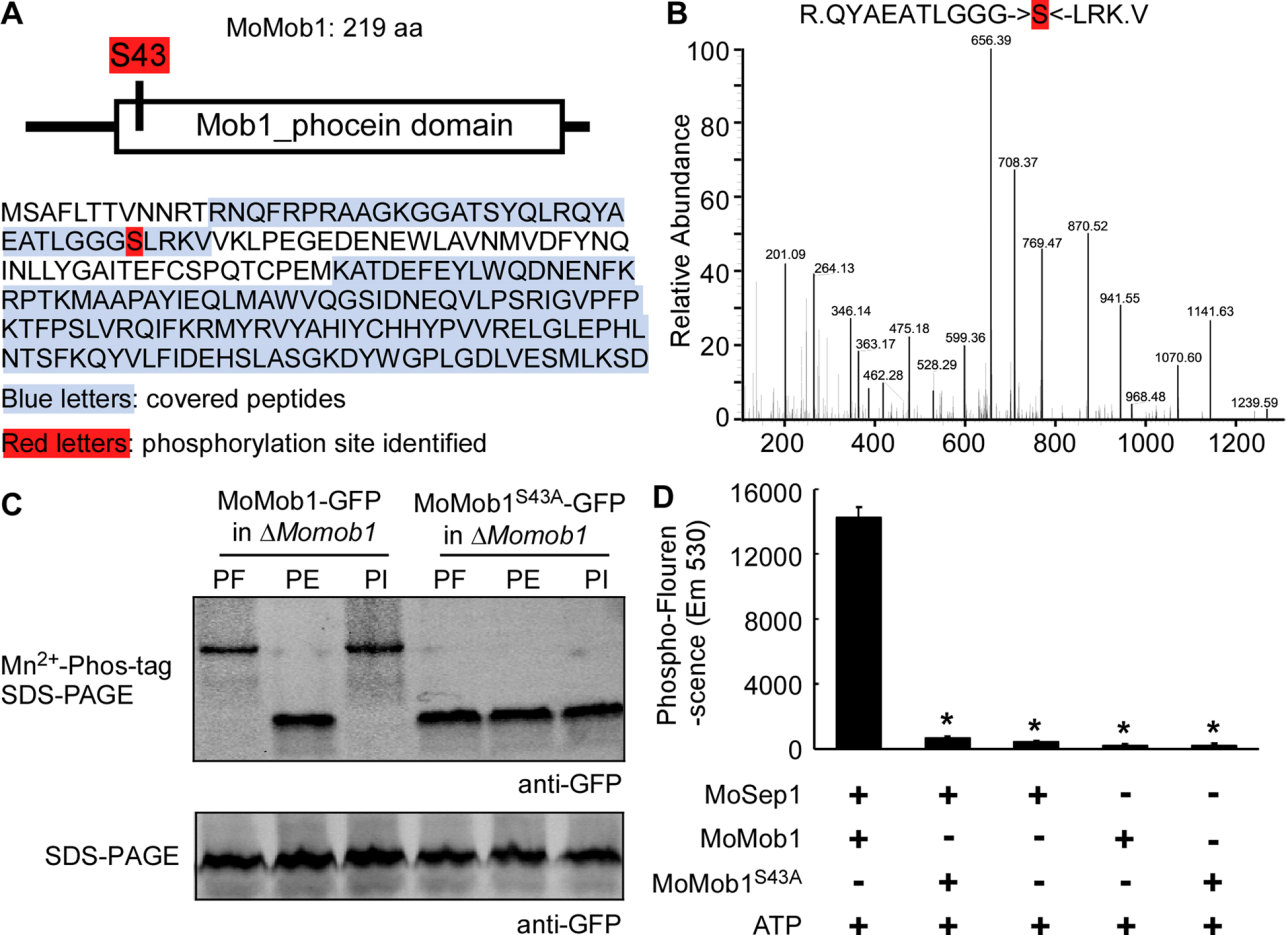
S43 of MoMob1 is the key phosphorylation site by MoSep1. **A** Comparison of MoMob1 phosphorylation sites in Guy11 and the Δ*Mosep1* mutant identified by LC-MS/MS analysis. **B** MoMob1 phosphorylation sites in Guy11 compared with the Δ*Mosep1* mutant expressing *MoMOB1* identified by LC-MS/MS analysis. **C** In vivo phosphorylation analysis of MoMob1 in the Δ*Momob1/MoMOB1*^*S43A*^ *and* Δ*Momob1/MoMOB1* strains. MoMob1-GFP proteins treated with phenylmethylsulfonyl fluorid (PF), phosphatase (PE) and phosphatase inhibitors (PI), and detected by the anti-GFP antibody. The phosphorylated-MoMob1 band is higher than controls when detected in the Mn^2+^-Phos-tag SDS-PAGE gel. **D** In vitro phosphorylation analysis by the fluorescence detection in tube (FDIT) method. Purified proteins of His-MoMob1, His-MoMob1^S43A^, and His-MoSep1 were used for kinase reactions in the presence of 50 μm ATP and dyed with the Pro-Q Diamond Phosphorylation Gel Stain. The fluorescence signal at 590 nm (excited at 530 nm) was measured in a Cytation3 microplate reader (Biotek, Winooski, VT, USA). Error bars represent the standard deviations from three independent experiments. Asterisks indicate statistical significance according to a Student’s test (*p* < 0.01)

Since MoSep1 directly phosphorylates MoMob1, we determined MoMob1 specific phosphorylation sites by MoSep1. We purified the MoMob1-GFP protein from the Δ*Mosep1* mutant and Guy11 and found that S43 residue is the only phosphorylated site in MoMob1-GFP isolated from Guy11 that was not present in the Δ*Mosep1* mutant (Fig. 3A and B). To verify this phosphorylation site, the constitutively unphosphorylated MoMob1^S43A^-GFP fusion protein was obtained and expressed in the Δ*Momob1* mutant strain. The phosphorylation level of MoMob1 in Δ*Momob1/MoMOB1*^*S43A*^ declined compared to the complemented strain Δ*Momob1/MoMOB1* (Fig. 3C). In addition, an in vitro phosphorylation assay using purified His-MoMob1^S43A^, His-MoMob1, and His-MoSep1 found lower levels of phosphorylation by MoSep1, in comparison to the wild-type MoMob1 (Fig. 3D). All these results suggested that MoMob1 S43 residue is essential for the phosphorylation of MoMob1 by MoSep1.

### MoSep1-dependent MoMob1 phosphorylation is important for vegetative growth, conidiation, and pathogenicity

**Fig. 4:**
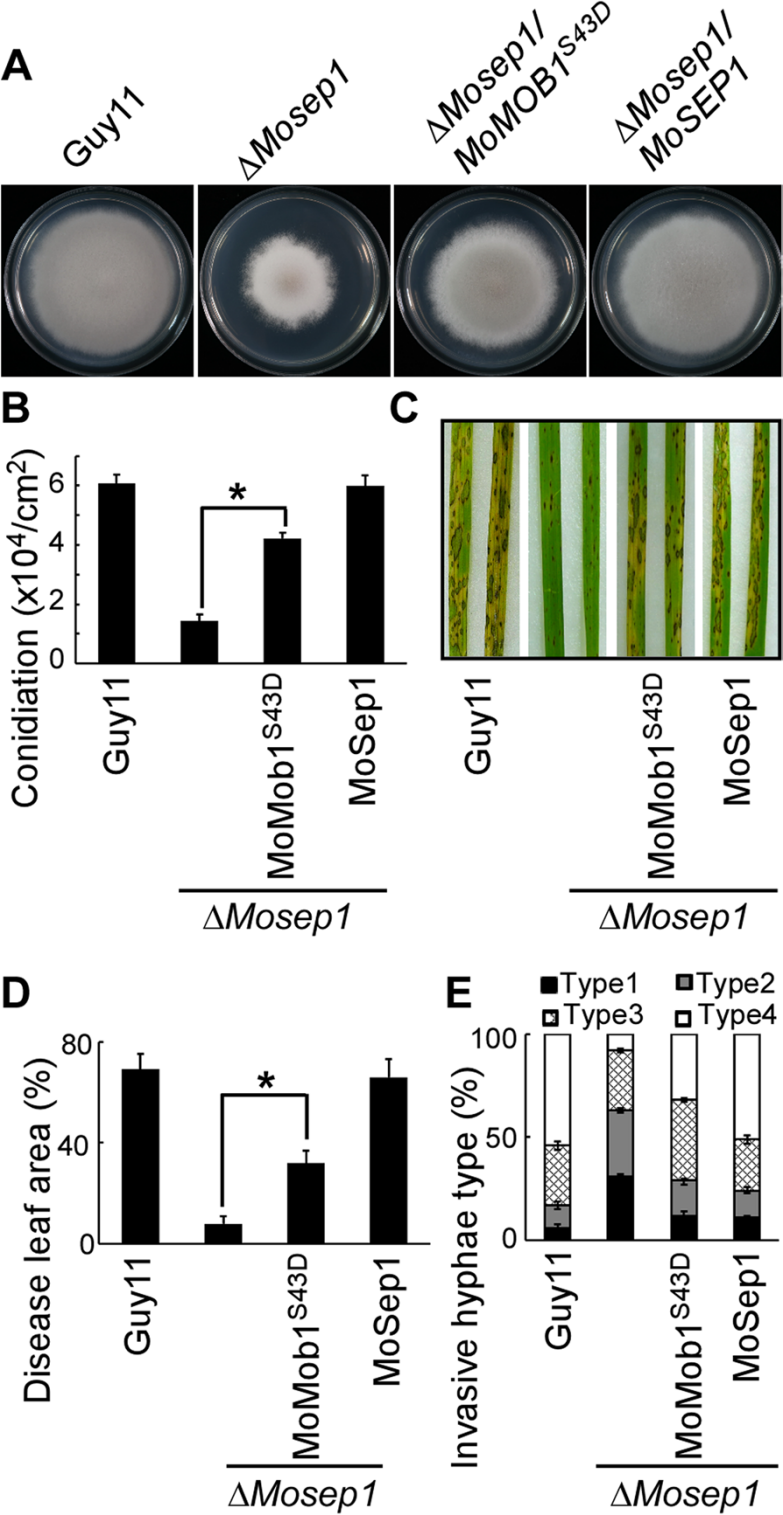
MoMob1^S43D^ phosphomimic mutation partly suppresses defects of *Mosep1* mutation. **A** Growth of the wild-type Guy11, mutant Δ*Mosep1* and Δ*Mosep1/MoMOB1*^*S43D*^, and complemented Δ*Mosep1/MoSEP1* strains on CM medium. **B** Statistical analysis of conidia production on SDC medium cultured at 28 °C for 7 days in the dark followed by 3 days of continuous illumination under fluorescent light. Error bars represent the standard deviations from three independent experiments. Asterisks indicate statistical significance according to a Student’s test (*p* < 0.01). **C** Pathogenicity analysis using rice spraying assays. Images were photographed at 7 dpi. **D** Diseased leaf area analysis. Data were presented as a bar chart showing the percentage of lesion areas. Error bars represent the standard deviations from three independent experiments. Asterisks denote statistical significance according to the Student’s test (*p* < 0.01). **E** Statistical analysis of infectious hyphae types (type 1, appressorium only; type 2, with primary invasion hyphal; type 3, secondary invasive hypha does not extend to neighboring plant cells; type 4, invasion hyphal extended into neighboring plant cells) on rice leaf sheaths. Rice leaf sheaths were inoculated with conidial suspensions and examined at 36 h post-inoculation (hpi). One hundred infectious hyphae were counted for each strain, and experiments were repeated three times. Error bars represent standard deviations

To investigate the role of MoSep1-dependent phosphorylation in *M. oryzae*, we examined the vegetative growth, conidiation, and virulence of the MoMob1^S43D^-GFP mutant strain. Results showed that MoMob1^S43D^-GFP suppressed the growth defects of the Δ*Μosep1* mutant, and the conidiation of Δ*Μosep1/MoMob1*^*S43D*^ was significantly increased compared with Δ*Μosep1*. MoMob1^S43D^-GFP could also restore the virulence defect of Δ*Μosep1* (Fig. 4).

**Fig. 5:**
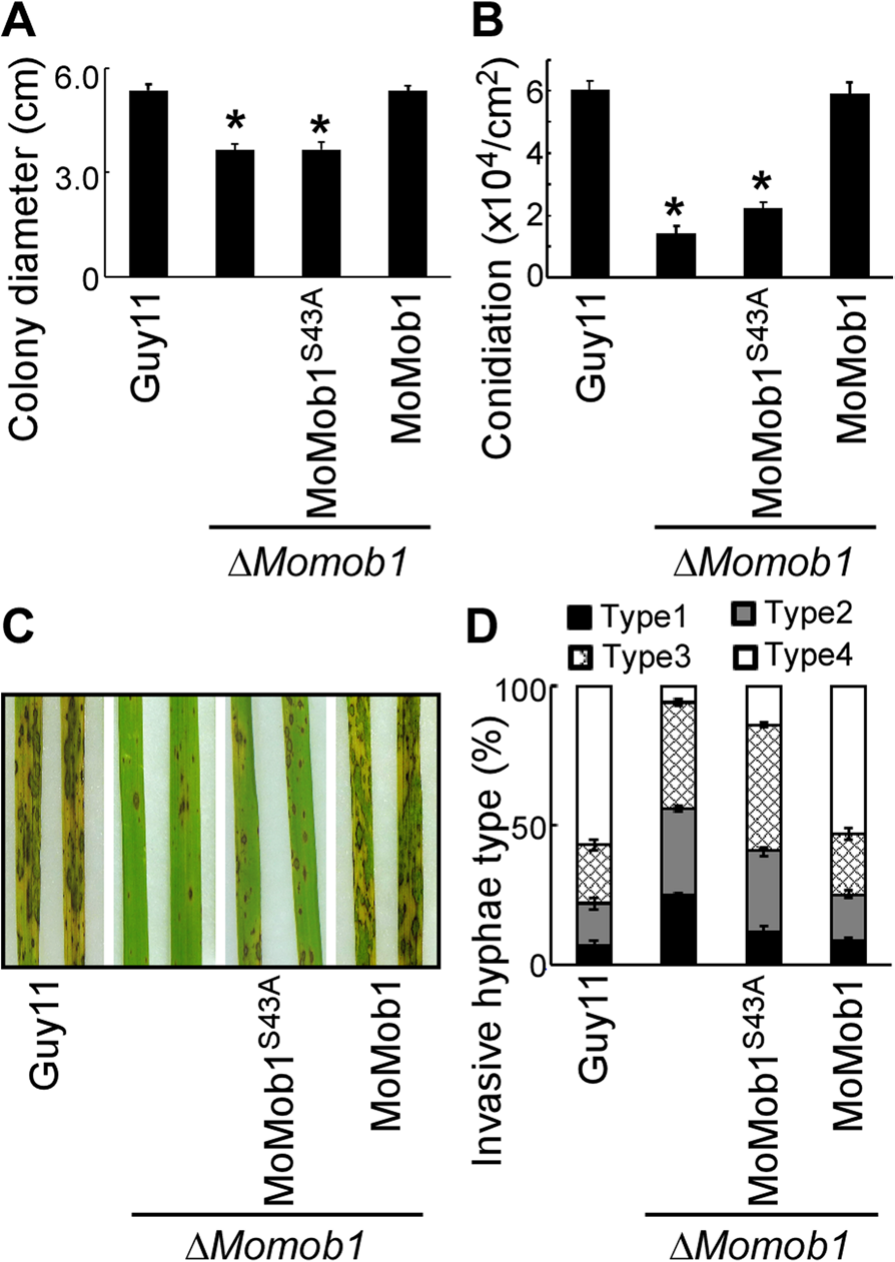
MoSep1-dependent MoMob1 phosphorylation is vital for vegetative growth, conidiation, and pathogenicity of *M. oryzae*. **A** Statistical analysis of colony diameters from wild-type Guy11, mutants Δ*Momob1* and Δ*Momob1/MoMOB1*^*S43A*^, and complemented strain Δ*Momob1/MoMOB1* on CM medium. Error bars represent standard deviations from three independent experiments. Asterisks indicate statistical significance according to a Student’s test (*p* < 0.01). **B** Statistical analysis of conidia production on SDC medium cultured at 28 °C for 7 days in the dark followed by 3 days of continuous illumination under fluorescent light. Error bars represent the standard deviations from three independent experiments. Asterisks indicate statistical significance according to a Student’s test (*p* < 0.01). **C** Pathogenicity analysis using rice spraying assays and images were photographed at 7 dpi. **D** Statistical analysis of infectious hyphae types (type 1, appressorium only; type 2, with primary invasion hyphal; type 3, secondary invasive hypha does not extend to neighboring plant cells; type 4, invasion hyphal extended into neighboring plant cells) on rice leaf sheaths. Rice leaf sheaths were inoculated with conidial suspensions and examined at 36 h post-inoculation (hpi). One hundred infectious hyphae were counted for each strain, and experiments were repeated three times. Error bars represent standard deviations

Given that activation of MoSep1-dependent MoMob1 phosphorylation could restore defects of the Δ*Μosep1* mutant, we carried out mutagenesis of MoMob1 by changing S43 to A43. The results showed that the growth rate of vegetative hyphae of Δ*Μomob1/MoMob1*^*S43A*^ was similar to that of the Δ*Μomob1* mutant. And in this phosphor-dead mutant, conidia were rarely produced, and virulence was significantly reduced compared with the Guy11 strain (Fig. 5). These results indicated that the MoSep1-dependent MoMob1 phosphorylation is essential for the development and pathogenicity of *M. oryzae*.

### MoSep1-dependent MoMob1 phosphorylation is important for MEN in *M. oryzae*

**Fig. 6:**
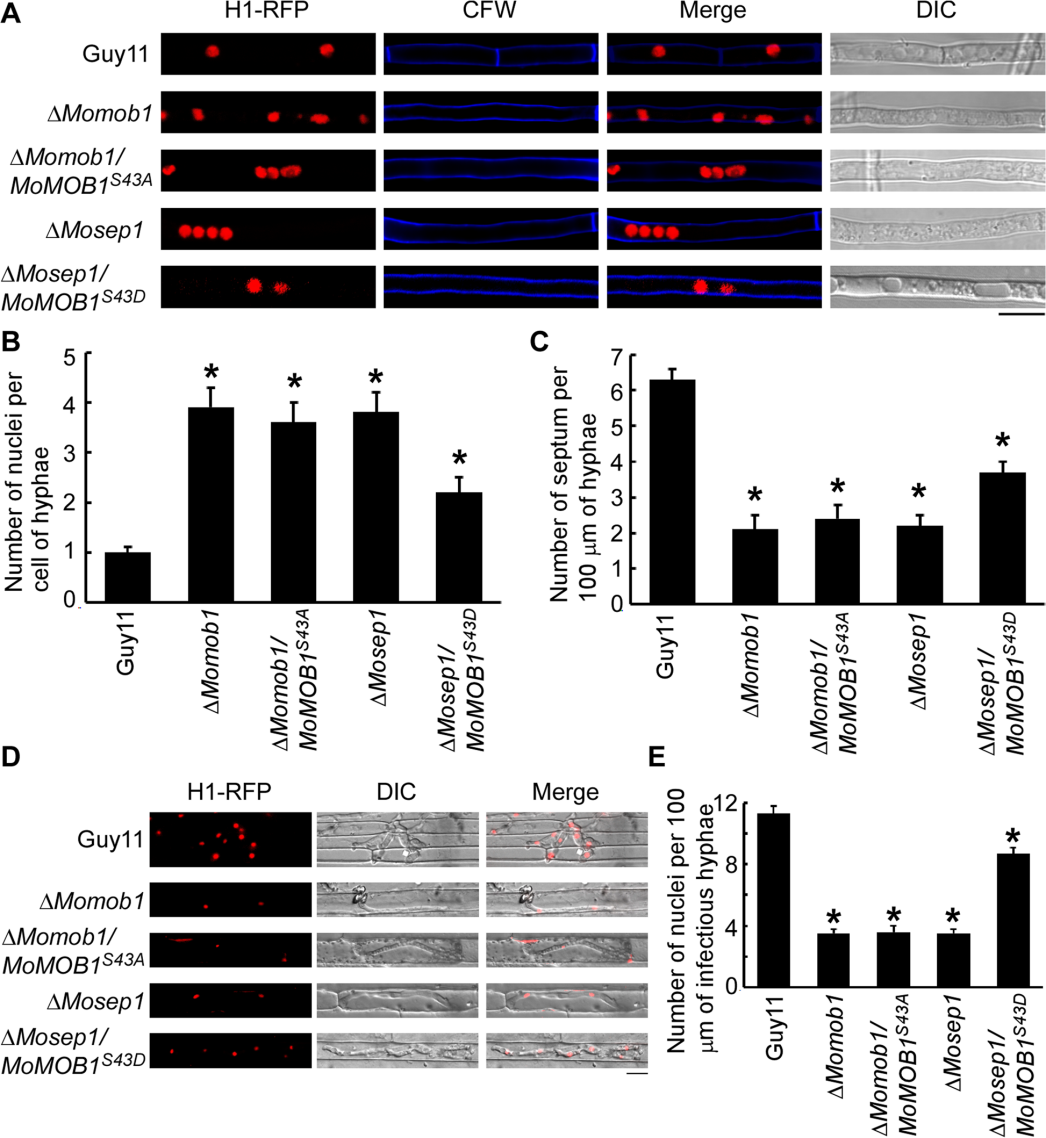
MoSep1-dependent MoMob1 phosphorylation is important for MEN signaling in *M. oryzae*. **A** Hyphae from Guy11, Δ*Momob1*, Δ*Momob1/MoMOB1*^*S43A*^, and Δ*Mosep1*, Δ*Mosep1/MoMOB1*^*S43D*^ strains expressing the H1-RFP construct were stained with CFW and examined by epifluorescence microscopy. Bar, 10 μm. **B** Statistical analysis of nucleus number in hyphae. Error bars represent the standard deviations. Asterisks indicate statistical significances (*p* < 0.01). **C** Statistical analysis of septum number per 100 μm hyphae. Error bars represent the standard deviations. Asterisks indicate statistical significances (*p* < 0.01). **D** Infectious hyphae of Guy11, Δ*Momob1*, Δ*Momob1/MoMOB1*^*S43A*^, and Δ*Mosep1*, Δ*Mosep1/MoMOB1*^*S43D*^ mutants expressing the H1-RFP construct examined by epifluorescence microscopy. Bar, 10 μm. **E** Statistical analysis of the number of nuclei per 100 μm infectious hypha in (**D**). Error bars represented the standard deviations. Asterisks denote statistical significances (*p* < 0.01)

As the constitutively activated MoMob1^S43^ was able to suppress defects of the Δ*Μosep1* mutant, we wondered if the phosphorylation of MoMob1 by MoSep1 was also involved in regulating the MEN pathway. To test this, the H1-RFP construct was transformed into Δ*Μosep1/MoMOB1*^*S43D*^ and Δ*Μosep1/MoMOB1*^*S43A*^ strains. When stained with Calcofluor White (CFW), more septa were observed in the Δ*Μosep1/MoMOB1*^*S43D*^ strain than in the Δ*Μosep1* mutant, but fewer septa in the ΔΜ*osep1/MoMOB1*^*S43A*^ strain than Guy11, suggesting that the activation of this key phosphorylation site dependent on MoSep1 is important for septum formation. In addition, normal hyphae have one nucleus per cell; however, after inactivation of the specific phosphorylation site on MoMob1, more than one nucleus was observed per hyphae cell (Fig. 6A-C). In addition, the infectious hyphae extension induced in the Δ*Mosep1/MoMob* ^*S43D*^ strain than that in the Δ*Mosep1* mutant, and exhibited 8–9 nuclei per 100 μm of infectious hyphae which were similar to wild type strain (Fig. 6D and E). These results further indicated the importance of the activation of MoMob1 by MoSep1 in cytokinesis and mitosis of *M. oryzae*.

**Fig. 7:**
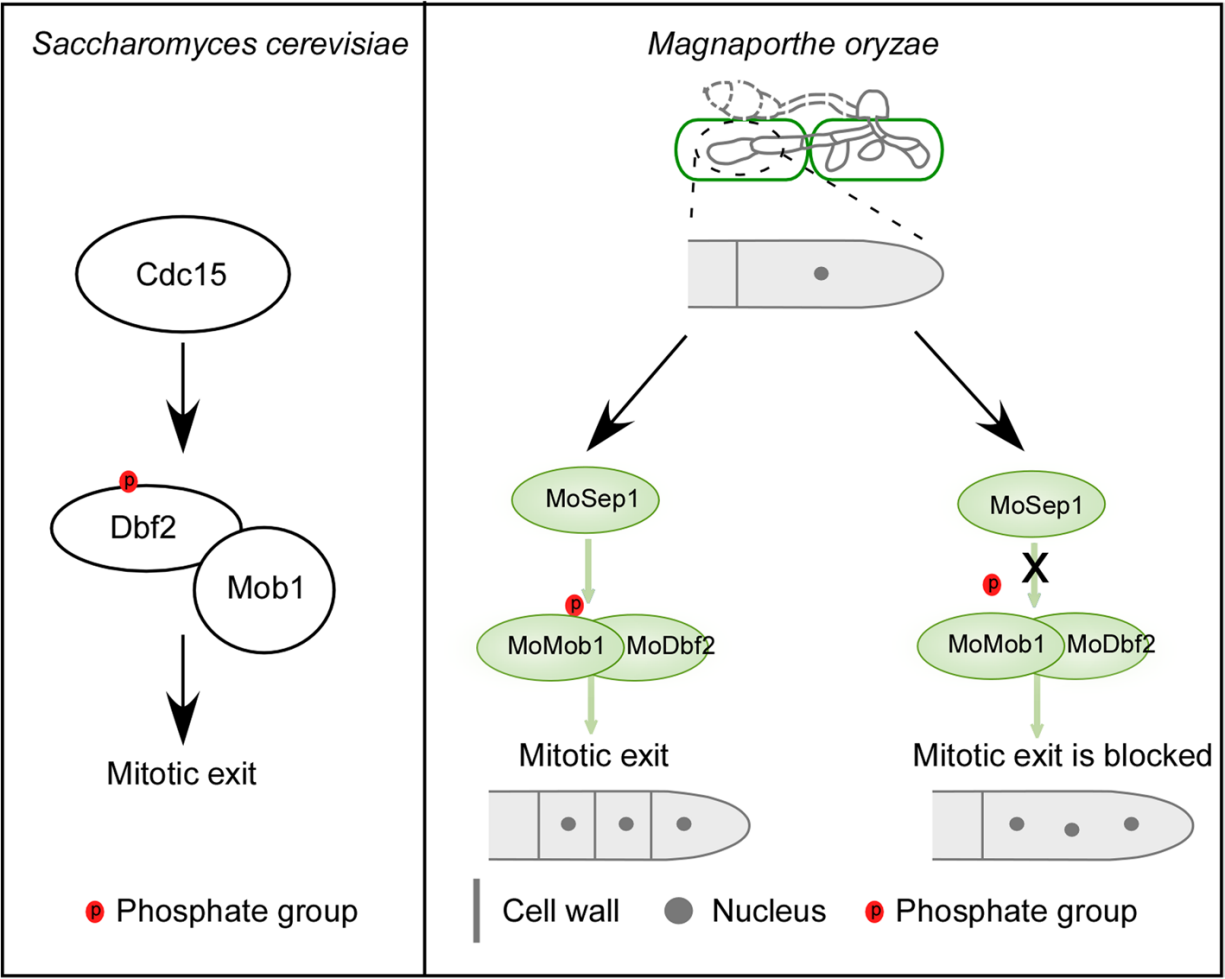
Comparison of mitotic exit network (MEN) signalling between *Magnaporthe oryzae* MoSep1-MoMob1-MoDbf2 and *Saccharomyces cerevisiae* Cdc15-Dbf2-Mob1. In the rice blast fungus *Magnaporthe oryzae*, the MEN pathway, consisting of conserved protein kinases MoSep1 and MoMob1-MoDbf2, is important in the development and pathogenicity of the fungus. *M. oryzae* Cdc15 homolog MoSep1 showed core functions in the MEN pathway important for growth, development, and pathogenesis. However, MoSep1 directly regulates MoMob1 phosphorylation, but not MoDbf1, in contrast to *S. cerevisiae*. In *M. oryzae*, when the phosphorylation of MoMob1 by MoSep1 is blocked, septa could not be normally formed and multiple nuclei per cell led to the disrupt of cell division

Collectively, we have found that phosphorylation levels of MoMob1 was significantly reduced in the Δ*MoSep1* mutant and that the S43 residue of MoMob1 is subject to MoSep1-dependent phosphorylation. Our results showed that MoSep1-dependent MoMob1 phosphorylation plays an important role in the growth, development, and pathogenesis of *M. oryzae* (Fig. 7). However, as the deletion of *MoSEP1* showed no significant effect on phosphorylation levels of MoDbf2, we speculated that MoSep1 does not regulate the phosphorylation of MoDbf2, and there might be other important kinases that regulate MoDbf2 and maintains the normal cellular activities.

## Discussion

Timely and correct cell division during mitosis is essential for vegetative growth, conidium germination, appressorium formation, and host penetration during *M. oryzae* infection (Dagdas et al. 2012; Ebbole 2007; Zhang et al. 2016). In the process of conidium germination to form appressorium, the nucleus on the distal side enters the germ tube allowing for timely replication of genetic materials and nuclear division (Fernandez and Orth 2018). Following this, one subnucleus moves to the young appressorium to form a diaphragm, and the other subnucleus returns to the original conidial cell. With the maturation of adherent cells, the inner wall of adherent cells is gradually covered by the melanin layer, and turgor pressures build up (Ryder and Talbot 2015). All of these processes require precise cell cycle regulation.

Eukaryotic organisms exhibit regulatory mechanisms to govern the entry and exit of cell division. During division, centrosomes produce microtubules to interact with centromeres and participate in spindle assembly. The MEN pathway is involved in the regulation of spindle directional control and cytokinesis. In *S. cerevisiae*, Cdc15, as an important nodal protein of the MEN pathway, is involved in regulating the activities of multiple proteins, and changes in organelle localizations strictly regulate mitosis exit. GTPase Tem1 is a molecular switch that activates Cdc15 and Dbf2-Mob1 (Hotz and Barral 2014). In addition to Tem1, Cdc5 also regulates the localization of Cdc15 to the spindle body (Rock and Amon 2011). The phosphorylation of the scaffold protein Nud1 by Cdc15 also contributes to the activation of Df2-Mob1 (Gruneberg et al. 2000; Rock et al. 2013). Moreover, Cdc15 recruits CDK to the spindle body in the early stage of anaphase, and CDK in turn negatively regulates the binding of Cdc15 to the spindle (König et al. 2010).

Similarly, *M. oryzae* Cdc15 homolog MoSep1 showed core functions in the MEN pathway important for growth, development, and pathogenesis. Here, we found that there are also different regulatory mechanisms between *M. oryzae* and *S. cerevisiae*. In *S. cerevisiae,* Cdc15 phosphorylate Dbf2 to activate the release of Cdc14, thus guiding cell division to exit and prepare for the next round of cell division. In *M. oryzae*, however, the phosphorylation of MoDbf2 showed no significantly affect in Δ*Mosep1* mutant, while the phosphorylation level of MoMob1 (another component of MoDbf2-MoMob1 complex) decreased significantly. Furthermore, we found that S43 residue of MoMob1 is the key phosphorylation site dependent on MoSep1, and it is important for vegetative growth, conidiation, and pathogenicity. These results indicate that although the MEN pathway is conservative in regulating cell division and exit in eukaryotes, it also has a relatively evolutionary specific regulatory mechanism in different species. And as the important MEN pathway protein kinase, MoSep1 also plays an important role in coordinating other signaling pathways during cell division. As our previous studies demonstrated, MoSep1 links the MEN pathway to Cell Wall Integrity (CWI) signaling through the phosphorylation of the CWI component MoMkk1 (Feng et al. 2021). MoSep1-dependent phosphorylation of MoMkk1 relieves the cell wall stress during cell division, providing new evidence on adapting to self-generated stress for growth. Therefore, further study of MoSep1 would facilitate studies of pathogenesis mechanisms of the blast fungus.

## Material and methods

### Strains and culture conditions

The *M. oryzae* Guy11 strain was used as a wild-type strain (WT) in this study. All strains were cultured on complete medium (CM) agar plates in the dark at 28°C, unless indicated otherwise (Kapoor 2017). Fungal mycelia grown in liquid CM for 1 day or 2 days at 28°C were harvested for microscopic observation and nucleic acid and protein extraction.

### Yeast two-hybrid (Y2H) assay

The constructs were generated by cloning cDNA of the MEN components *MoSEP1*, *MoDBF2*, and *MoMOB1* genes into pGBKT7, and into pGADT7 and pGBKT7. The resulting prey and bait constructs confirmed by DNA sequencing were transformed in pairs into the yeast strain AH109. Transformants were identified as described previously (Li et al. 2019).

### Phosphorylation analysis

The MoDbf2-GFP and MoMob1-GFP fusion constructs were transferred into Guy11 and the Δ*Mosep1* mutant. Total proteins extracted from mycelia were resolved on 8% SDS-PAGE gel prepared with 50 μm acrylamide-dependent Phos-tag ligand and 100 μm MnCl_2_, as previously described (Kapoor 2017). Gel electrophoresis was performed with a constant voltage of 80 V for 6–8 h. Gels were soaked in transfer buffer with 5–10 mM EDTA for 15 min three times before transferring, followed by transfer buffer without EDTA for another 10 min. Protein transfer from the Mn^2+^-phos-tag™ acrylamide gel to the PVDF membrane was performed for ~ 48 h (depending on different proteins) at 80 V at 4°C, and then the membrane was analyzed by Western blotting analysis using the anti-GFP antibody (Feng et al. 2021).

### Mass spectrometric analysis

To identify phosphorylation sites of targeted proteins, total proteins were extracted from MoMob1-GFP/Guy11 and MoMob1-GFP/ΔΜ*osep1* transformants. Approximately 30 μl of anti-GFP beads was added into 1 ml diluted total protein samples. Following similar purification steps as above, the eluted proteins were neutralized and separated on a 10% SDS-PAGE gel. The gel bands corresponding to the targeted proteins were excised from the gel and subject to mass spectrometry analysis as described previously (Li et al. 2019).

### In vitro phosphorylation analysis

The His-MoSep1, His-MoMob1, and His-MoMob1^S43A^ were expressed in *Escherichia coli* BL21-CodonPlus (DE3) cells and purified. A rapid Fluorescence Detection in Tube (FDIT) method using Pro-Q Diamond Phosphorylation Gel Stain was used to analyze protein phosphorylation. First, 2 μg MoMob1 (or the unphosphorylated mutations) was mixed with MoSep1 in a kinase reaction buffer (100 mM PBS, 1 mM ascorbic acid, pH 7.5, and 10 mM MgCl_2_), with 50 μM ATP at 25°C for 1 h. Ten folds of cold acetone was then added to terminate the reaction. Casein was homogenized and suspended in Mili-Q water at a concentration of 0.2 μg/ml to stain the proteins. Briefly, 100 μl of Pro-Q Diamond was mixed with 10 μl of casein, and the mixture was kept in the dark for 1 h. Ten folds of cold acetone were then added, and the mixture was allowed to incubate overnight at − 20°C. Proteins were precipitated by centrifugation at 14,000 rpm for 1 h at 4°C. The supernatants were discarded, and protein pellets were washed twice using 500 μl cold acetone. The pellets were dissolved in 200 μl of Mili-Q water and transferred to a 96 well plate. The fluorescence signal at 590 nm (exited at 530 nm) was measured using a Cytation 3 microplate reader (Yin et al. 2020).

### Epifluorescence microscopy and nucleus staining and CFW staining

*M. oryzae* cells, including hyphae, conidia, and appressorium expressing fluorescent fusion proteins, were incubated under appropriate conditions. The constructs, including H1-RFP and other phosphorylation mutations, were transformed into testing strains. Epifluorescence microscopy was performed using a Zeiss LSM710 microscope. DAPI was added to the cell preparations at a concentration of 1 μg/ml for 5 min (room temperature and in darkness). For CFW staining, mycelia were stained with 10 μg/ml for 5 min in the dark to visualize nuclei (Feng et al. 2021).

### Plant infection assays

Conidia were adjusted to 5 × 10^4^ spores/ml in a 0.2% (w/v) gelation solution, and 5 ml was sprayed on 2-week-old rice seedlings (*Oryza stative* cv. CO39). Inoculated plants were kept in a chamber at 25°C under 90% humidity for the first 24 h, followed by a light/dark cycle for 4–7 days.

## Data Availability

All data generated or analyzed during this study are included in this published article.

## Abbreviations

Cdks: Cyclin-dependent kinases
CFW: Calcofluor White
CWI: Cell Wall Integrity
CycB: Cyclin B
MEN: Mitotic exit network
MS: Mass spectrometry
SPB: Spindle pole body
SPOC: Spindle position checkpoint
